# Probiotic Potential and Safety Properties of *Lactobacillus plantarum* from Slovak Bryndza Cheese

**DOI:** 10.1155/2013/760298

**Published:** 2013-09-04

**Authors:** Anna Belicová, Mária Mikulášová, Roman Dušinský

**Affiliations:** ^1^Department of Molecular Biology, Faculty of Natural Sciences, Comenius University, Mlynská Dolina, 842 15 Bratislava, Slovakia; ^2^Department of Genetics, Faculty of Natural Sciences, Comenius University, Mlynská Dolina, 842 15 Bratislava, Slovakia

## Abstract

One hundred and twenty-five acid-resistant presumptive lactobacilli were isolated from Slovak Bryndza cheese and screened for their antimicrobial activity against eight bacterial pathogens using spot agar assay. Out of twenty-six *Lactobacillus* strains with strong inhibition activity, twenty were identified as *Lactobacillus plantarum* and six as *Lactobacillus fermentum*. The most active eleven *L. plantarum* isolates were further characterized *in vitro* for some probiotic and safety properties. Only three isolates K10, K21, and ZS07 showed the ability to grow over 50% in the presence of 0.3% bile. Strong deconjugation efficiency was determined for CK06 and K21. The highest **β**-galactosidase activity was shown in isolates ZS11, B01, CK06, and ZS07. Only three of the strains had the ability to produce tyramine: CK06, LM1, and ZS11. Strains K09, K21, ZS11, and ZS15 were susceptible to all tested antibiotics. Analysis of the results confirmed the *L. plantarum* isolates ZS07 and K21 as the most suitable for probiotic use, due to their desirable probiotic and safety characteristics.

## 1. Introduction

Probiotics are defined as live microorganisms, which, when consumed in appropriate amounts, result in a health benefit to the host [1]. Species of the genera *Lactobacillus* and *Bifidobacterium* are among the lactic acid bacteria most commonly used as probiotics in animal feeds and human foods. They are “generally regarded as safe” (GRAS status) according to The American Food and Drug Administration due to their long history of safe use in fermented foods and their presence in the normal intestinal and urogenital microbiota of humans. Several species, including *L. plantarum *and *L. fermentum*, have received a Qualified Presumption of Safety (QPS) status given by European Food Safety Authority (EFSA). According to the recommendations for evaluation of probiotics, putative probiotic strains should be screened for their essential functional properties (resistance to gastric acidity and bile salts, production of antimicrobial compounds, the ability to modulate immune responses, and adhesion to gut tissues) and safety properties such as antibiotic resistance and production of biogenic amines in *in vitro* tests. Other recommendations include the absence of hemolytic activity and transferable antibiotic resistance of the strains, where the safety should be proven in animal models [1].

Slovak Bryndza cheese is a natural, spreadable cheese with characteristic odour and taste, made using the traditional method: by milling a lump of matured ewes' cheese, or by milling a mixture of ewes' lump cheese and cows' lump cheese. Bryndza cheese includes several predominant lactic acid bacteria (LAB) belonging to the genera *Lactobacillus* spp., *Enterococcus* spp., *Lactococcus* spp., and *Streptococcus* spp. [2, 3]. *L. plantarum* are ubiquitous lactic acid bacteria that are detected in environments such as food (dairy products, fermented meat, vegetables, fruits, and beverages), respiratory, gastrointestinal, and genital tracts of humans and animals, and in sewage and plant material.

Several *L. plantarum* isolates proved the ability to survive gastric transit and to colonize the intestinal tract of humans and other mammals [4, 5]. Certain studies showed that amongst the other effects, consumption of *L. plantarum* reduced carriage of faecal *Enterobacteriaceae*, decreased certain risk factors for coronary artery disease, and resulted in a dose-dependent reduction in the symptoms of IBS [6]. It seems that, in the research for strains with probiotic potential, food might also be a good source of suitable isolates for finding new probiotic strains for functional food products. The traditional recommendation that probiotic strains for humans should come from humans (species-specificity criterion) is becoming mitigated, because at present, several probiotic products include nonstarter LAB (NSLAB) such as *L. paracasei* and *L. plantarum*. These food and health products containing probiotic strains of *L. plantarum* are commercially available [6].

The aims of our research were (1) to characterize *L. plantarum* strains isolated from Bryndza cheese and (2) to select the most suitable strains for use as probiotics, according to their functional and safety attributes, including antagonistic activity against pathogens, bile resistance, bile salt deconjugation, *β*-galactosidase activity, antibiotic resistance, and production of biogenic amines.

## 2. Materials and Methods

### 2.1. Sampling and Isolation of Acid-Resistant Lactobacilli

A total of 125 presumptive acid tolerant lactobacilli were isolated from Slovak Bryndza cheese. The Bryndza samples were obtained from five commercial producers, namely, Brezňan (B), Červený Kameň (CK), Kluknava (K), Liptovský Mikuláš (LM), and Zvolenská Slatina (ZS). Bryndza from Brezňan was made from fresh ewes' lump cheese, from unpasteurized ewes' milk. Other Bryndza samples, namely, LM and ZS, were made from fresh ewes' lump cheese (from pasteurized ewes' milk) and cows' lump cheese made from pasteurized cows' milk, while CK and K were made from ewes' lump cheese stored for several months and cows' lump cheese made from pasteurized cows' milk, where the ewes' lump cheese represented more than 50% of the mixture in dry substance.

LAB were screened for their resistance to low pH. Briefly, the cheese sample was emulsified in sterile 2% (w/v) trisodium citrate at 45°C for 3 min, and cells were harvested by 5 min centrifugation at 12000 ×g. The pellet was washed twice with 1/4 Ringers solution and finally resuspended in MRS medium (pH 2.0 adjusted by 1 N HCl). Bacteria were cultivated at 37°C for 3 h then serially diluted in sterile saline solution and plated in triplicate on the MRS agar. The plates were incubated anaerobically for 48 h at 37°C (Bugbox, Ruskinn Technology, UK). Twenty-five randomly selected colonies of Bryndza cheese samples from each producer were purified by two subsequent subcultures and then submitted to microscopic examination, Gram staining, and catalase test. Colonies of catalase-negative, Gram-positive rods were presumed to be lactobacilli. They were stored in MRS containing 20% glycerol at −80°C.

### 2.2. Screening for Antagonistic Activity

Antagonistic activity of isolates was evaluated as described elsewhere [7]. Overnight cultures of the isolates were spotted onto the surface of agar plates (MRS-0.2 with 1.2% agar) and incubated anaerobically for 24 h at 37°C (Bugbox, Ruskinn Technology, UK). 100 *μ*L 18 h cultures of indicator strains were inoculated into 7 mL of soft BHI agar (containing 0.7% agar) and poured over the plate on which the producer was grown. After aerobic incubation for 48 h at 37°C, the plates were checked for inhibition zones. Inhibition was scored positive if the width of the clear zone around the colonies of the producer strain was 1 mm or larger. The following pathogens and opportunistic pathogens were used as indicator strains: *Listeria monocytogenes* CCM 4699; *Staphylococcus lentus *CCM 3472; *Acinetobacter calcoaceticus *CCM 4503; *Sphingomonas paucimobilis *CCM 3293; and* Salmonella enterica *subsp. *enterica*, serovar Typhimurium strain TA100 CCM 3812 from the Czech Collection of Microorganisms, Brno, Czech Republic,* Enterococcus faecalis* V583 from the University of Oklahoma, USA, *Staphylococcus aureus* SSV25, and *Staphylococcus epidermidis* SSV30 (from our collection).

The strains exhibiting antagonistic activities were further tested for their activity of cell-free neutralised supernatants (CFNS) using the agar spot test method of Uhlman et al. [8]. Briefly, CFNS were obtained from cultures grown in MRS broth for 18 h at 37°C. After centrifuging the culture at 12000 ×g for 15 min, the supernatant was adjusted to pH 6.5 with NaOH and heated at 100°C for 5 min. The supernatants were tested against the same indicator strains used previously.

### 2.3. Identification of Selected Isolates

Twenty-six putative lactobacilli were identified at species level by the API 50 CH System and 50 CHL medium (Bio-Mérieux, France), according to the manufacturer's instructions. Results were recorded after 2 days of incubation at 37°C and evaluated with the identification software ApilabPlus provided by Bio-Mérieux.

#### 2.3.1. Preparation of Cell Lysates

Two colonies of isolates were suspended in 50 *μ*L of Tris-HCl-EDTA-saline (pH 8.0). The bacterial suspension was incubated for 10 min at 95°C and centrifuged at 18600 ×g for 2 min, and the obtained supernatant served as the PCR template.

#### 2.3.2. PCR Identification

The isolates were identified using the species-specific primers for *L. plantarum* LbP11 (5′ AATTGAGGCAGCTGGCCA 3′) and LbP12 (5′ GATTACGGGAGTCCAAGC 3′) [9] and primers for *L. fermentum* Lfer3 (5′ ACTAACTTGACTGATCTACGA 3′) and Lfer4 (5′ TTCACTGCTCAAGTAATCATC 3′) [10]. Primers Lb1 (5′ AGAGTTTGATCATGGCTCAG 3′) and Lb2 (5′ CGGTATTAGCATCTGTTTCC 3′) were employed as positive control of PCR with LbP11 and LbP12 primers. All primers used in this study were obtained from Invitrogen (USA). PCR amplification was carried out in 25 *μ*L reaction containing 0.5 *μ*L dNTP (10 *μ*M in each dNTP), 0.75 *μ*L primer (10 pM), 0.42 *μ*L cell lysate, 2.5 *μ*L reaction buffer (10x), 0.17 *μ*L Taq DNA polymerase (5 U/*μ*L, GeneCraft, Germany), and 18.9 *μ*L deionized water. PCR reactions were performed with PTC-100 Peltier thermal cycler (MJ research, USA). The mixture was denatured for 5 min at 95°C and cycled 35 times at 94°C for 30 s, 54°C for 1 min (55°C for *L. fermentum*), and 72°C for 1 min, followed by a final 10 min extension at 72°C. PCR products were separated by electrophoresis on a 1.5% agarose gel stained with ethidium bromide, visualised under UV light. The size of each PCR product was determined by comparison with the 100 bp DNA ladder (Fermentas, Latvia). *L. plantarum* CCM 4281 and *L. fermentum* CCM 91 were used as controls for species identification.

Genetic diversity of isolates was determined by (GTG)_5_-PCR according to Versalovic et al. [11] using the single oligonucleotide primer (5′GTGGTGGTGGTGGTG 3′). The rep-PCR profiles were analyzed by the software Bio-1D (Vilber Lourmat, France). The similarity among digitized profiles was calculated using the Jaccard coefficient, and an average linkage (UPGMA) dendrogram was derived.

### 2.4. Bile Resistance

Survival of isolates in the presence of bile was determined according to the method of Vinderola and Reinheimer [12]. Isolates were inoculated (2% w/v) into MRS broth with 0.3%, 0.5%, or 1% (w/v) of bile (Sigma-Aldrich, USA). After 24 h cultivation at 37°C, *A*_560 nm_ was measured and compared to a control culture (without bile salts). The results were expressed by the percentage of growth (*A*_560 nm_) in the presence of bile salts compared to the control.

### 2.5. Bile Salt Deconjugation

The isolates were streaked on the bile salt plates, using MRS agar with 0.5% (w/v) of sodium salts (Sigma-Aldrich, USA) of taurocholic acid (TC), taurodeoxycholic acid (TDC), glycocholic acid (GC), and glycodeoxycholic acid (GDC), and these were anaerobically incubated at 37°C for 72 h. The ability of the isolates to deconjugate bile salts was declared by formation of precipitated bile acid around colonies—an opaque halo [13].

### 2.6. *β*-Galactosidase Activity

*β*-galactosidase activity of whole cells was determined according to the method of Miller [14], as modified by Vinderola and Reinheimer [12]. The *β*-galactosidase activity with o-nitro-*β*-D-galactopyranoside (Sigma-Aldrich, USA) as a reaction substrate was determined in cultures inoculated into lactose-MRS broth. After the reaction, optical densities at both 420 and 560 nm were determined, and *β*-galactosidase activity was calculated (Miller units) as follows: 1000 × [*A*_420_ − (1.75 × *A*2_560_)/(15 min × 1 mL × *A*1_560_)], where *A*1_560_ was the absorbance just before assay, and *A*2_560_ was the absorbance value of the reaction mixture.

### 2.7. Antibiotic Susceptibility Testing

The minimum inhibitory concentration for the 11 *L. plantarum* strains was determined by a broth microdilution test. Individual colonies were suspended in 5 mL sterile saline solution to a turbidity of 1 in the McFarland scale and further diluted 500-fold in LSM medium (Iso-sensitest broth : MRS, 9 : 1). Fifty *μ*L of the diluted bacterial suspensions was added to each well of manually premade MIC microtiter test plates (containing the different antibiotic test concentrations in each 50 *μ*L volume of LSM broth per well). The antibiotics were tested in the concentration ranges (mg/L): ampicillin (0.032–16), gentamicin (0.5–256), kanamycin (2–256), erythromycin (0.016–16), clindamycin (0.032–16), tetracycline (0.12–64), and chloramphenicol (0.12–64). Plates were incubated at 37°C for 24 h. The MICs were determined to be the lowest antibiotic concentration that inhibited visible bacterial growth, as measured turbidimetrically (*A*_650 nm_) by a microplate reader (Varioskan Flash, Thermo Scientific, Finland). Susceptible and resistant strains were distinguished according to the breakpoints (cutoff values) reported by EFSA [15]. Accordingly, strains showing MICs higher than the respective cutoff values were considered as resistant.

### 2.8. Production of Biogenic Amines

Biogenic amine production was examined on decarboxylating medium plates containing 2% L-histidine-monohydrochloride, L-tyrosine disodium salt, or L-ornithine monohydrochloride (Sigma-Aldrich, USA) as described by Joosten and Northolt [16]. Isolates were twice subcultured in decarboxylating broth supplemented with the corresponding amino acid precursor (1 g/L) and 1 mg/L pyridoxal 5-phosphate and incubated at 37°C for 24 h. 1 *μ*L of each culture was spotted on the decarboxylating agars, and plates were then incubated anaerobically (Bugbox, Ruskinn Technology, UK) at 37°C for 72 h. Beside the amine production on all media, a purple halo was interpreted as a positive reaction, except decarboxylation media containing tyrosine, where the positive response was presented by a clear halo surrounding the colonies. The experiments were performed three times.

## 3. Results and Discussion

A collection of 125 presumptive acid-resistant lactobacilli was isolated from Bryndza cheese samples from five commercial distributors. All isolates were selected based on their ability to survive 3 h cultivation at pH 2.0 and their positive Gram reaction, negative catalase reaction, and their rod shape (data not shown). The resistance to low pH is an important selection criterion for probiotic microorganisms, because gastric juice in the stomach destroys most microorganisms ingested. Burns et al. [17] and Jamaly et al. [18] also documented that all tested strains were able to tolerate three hours of acid exposure at pH 2.0 with only slow reduction of viability. Many other studies have confirmed that the exposure of *Lactobacillus* strains to pH values of 2.5–4.0 does not influence their survival rate, but it dropped at lower pH values [19–21]. The ability of lactobacilli to survive the passage through media with physiological pH of 2.0 to 3.0 (to mimic the stomach environment) was reported to be variable and strain dependent, but with a survival rate of approximately 85%, which is very significant for the probiotic field [22, 23].

All isolates were evaluated for their inhibitory activity towards selected Gram-positive and Gram-negative bacteria. In the agar spot test, the *Lactobacillus* isolates demonstrated different inhibitory activities when tested against indicator strains, while the zone of inhibition ranged from 1 mm to 5 mm (Table 1). 93% of *Lactobacillus *isolates displayed antimicrobial activity against *L. monocytogenes*. *S. lentus* and *E. faecalis* were inhibited by 82% and 80%, respectively. 86% of isolates showed antimicrobial activity against *S. aureus* and 79% against *S. epidermidis*. Many of the strains (93%) showed inhibitory activity against *S. enterica*. *S. paucimobilis* was inhibited in 68% of strains. Only 52% of *Lactobacillus* isolates exhibited activity against *A. calcoaceticus*.

**Figure 1:**
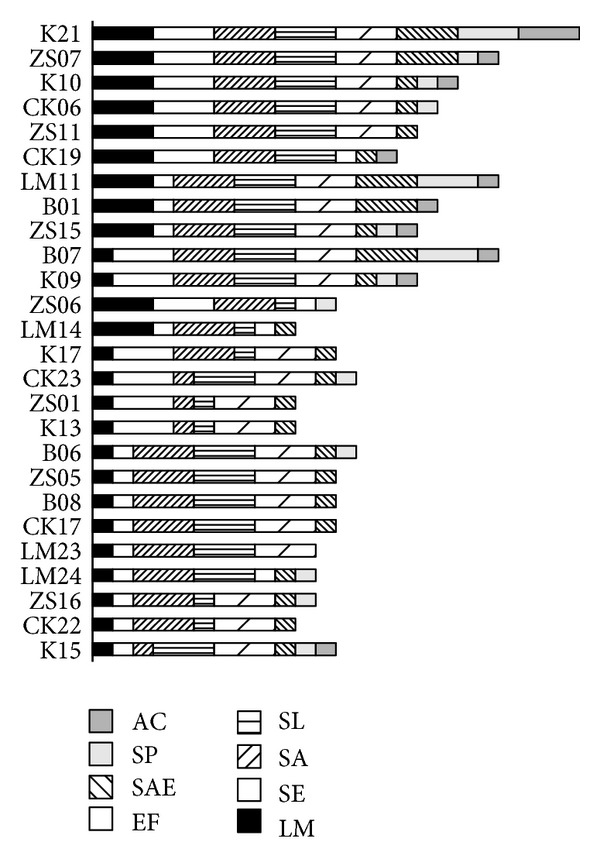
The inhibitory activity of the most active predictive lactobacilli isolates against the indicator strains. LM: *Listeria monocytogenes*; SA: *Staphylococcus aureus*; SE: *S. epidermidis*; SL: *S. lentus*; EF: *Enterococcus faecalis*; SAE: *Salmonella enterica*; SP: *Sphingomonas paucimobilis*; AC: *Acinetobacter calcoaceticus*. Long bars: inhibition zone = 1–5 mm and short bars: inhibition zone < 1 mm.

A total of 26 isolates were found to produce strong inhibition zones (zone of inhibition of more than 2 mm up to 5 mm) against at least two indicator strains (Figure 1). Twenty-two out of 26 strains of lactobacilli inhibited *S. aureus* and *E. faecalis*. Nineteen isolates inhibited *S. lentus*, thirteen *S. epidermidis*, eleven *L. monocytogenes*, five *S. enterica*, three *S. paucimobilis*, and one *A. calcoaceticus*. The isolate K21 displayed strong antimicrobial activity against all indicator strains. The most active lactobacilli (CK06, CK19, B01, B07, K09, K10, LM11, ZS07, ZS11, and ZS15) inhibited 4 or more indicator strains altogether. Variability was also found with respect to the susceptibilities of different indicator strains to lactobacilli. *S. paucimobilis* and *A. calcoaceticus* were the least susceptible pathogens, while *S. aureus* and *E. faecalis* exhibited the highest susceptibility to *Lactobacillus* isolates. None of the supernatants of the presumptive *Lactobacillus* strains at pH 6.5 inhibited the growth of the pathogens tested, using the spot assay (data not shown).

The antimicrobial activity observed could be explained by the production of organic acids, because the heated and neutralized cell-free supernatant of the producer culture did not exhibit any antimicrobial activity when compared to live cells in the spot assay. The inhibitory effect of hydrogen peroxide was excluded due to incubation of the plates under anaerobic conditions. Lactobacilli may antagonize pathogens by several mechanisms which involve production of antimicrobial compounds such as lactic acid, acetic acid, hydrogen peroxide, and bacteriocins, competition for substrate, and coaggregation with pathogens [24–27]. Many works documented that the production of organic acids is the main mechanism mediating the antimicrobial activity of the lactobacilli [28–30]. Furthermore, the degree of inhibition is specific to a particular strain and depends on the amounts of organic acids produced [31]. *Lactobacillus* isolates showed antilisterial activity and inhibited Gram-positive bacteria better than Gram-negative bacteria, which is in agreement with the findings of other studies [28, 32].

Based on the biochemical profile obtained by ApilabPlus software, CK06, CK19, CK22, B01, B06, B07, B08, K09, K10, K013, K015, K21, LM11, LM14, LM24, ZS01, ZS05, ZS07, ZS11, and ZS15 isolates were identified as *L. plantarum* and CK17, CK23, K017, LM23, ZS06, and ZS16 isolates as *L. fermentum*.

PCR amplification with species-specific primers according to Quere et al. [9] produced a DNA fragment corresponding in size to *L. plantarum* (250 bp) for isolates CK06, CK19, CK22, B01, B06, B07, B08, K09, K10, K013, K015, K21, LM11, LM14, LM24, ZS01, ZS05, ZS07, ZS11, and ZS15. Other isolates amplified a 192 bp product corresponding to* L. fermentum* (CK17, CK23, K017, LM23, ZS06, and ZS16). Results of biochemical (using API 50CHL—from Bio-Mérieux, France) and molecular identification of lactobacilli species were in 100% agreement. All identified lactobacilli were typized using (GTG)_5_ fingerprinting (data not shown). Isolates exhibited different (GTG)_5_ fingerprinting profiles (similarities less than 95%) and were considered to be genetically unrelated.

Twenty isolates were identified as *L. plantarum* and six as *L. fermentum*. *L. plantarum *belongs to the nonstarter LAB (NSLAB) and represents the majority of NSLAB found in most ripened cheeses [33]. Together with *L. brevis*, *L. parabuchneri*, *L. helveticus*, *L. paracasei*, *L. fermentum*, and *L. pentosus*, it constitutes the characteristic lactobacilli flora in Bryndza cheese [3]. The ability to survive low pH and broad-spectrum antagonism action due to organic acids is confirmed in many strains of *L. plantarum* and *L. fermentum *[4, 5], which is consistent with our results. Also Tejero-Sarinena et al. [31] observed the highest antimicrobial activity of *L. plantarum* in comparison with *L. fermentum*. This effect could be explained by a different pathway of the production of lactic acid by homofermentative and heterofermentative lactobacilli.

In view of our results, 11 isolates CK06, CK19, B01, B07, K09, K10, K21, LM11, ZS07, ZS11, and ZS15 which inhibited the growth of 4 or more indicator microorganisms with diameter of inhibition zone between 2 and 5 mm were selected for further studies (bile resistance, bile salt deconjugation, *β*-galactosidase activity, antibiotic susceptibility testing, and production of biogenic amines).

Survival of isolates in the presence of 0.3%, 0.5%, and 1% bile is shown in Table 2. The isolates displayed varying ability to grow in the presence of bile. All isolates, with the exception of K09, were able to grow in the presence of high concentrations (1%) of bile salts. Four isolates (B07, K10, K2, and ZS07) showed the highest bile resistance in the presence of 0.3% bile (over 50% compared to growth control). On the other hand, the highest percentage of survival in the presence of 0.5% and 1% bile was confirmed for CK19 (29% and 18%, resp.).

Ingested microorganisms are exposed to numerous environmental extremes in the human GIT. One of them is exposure to bile. Therefore, the ability of pathogens and commensals to tolerate bile is likely to be important for their survival and subsequent colonization of the GIT [34]. Survival of the majority of isolates from Bryndza decreased with increasing bile salt concentration, from 0.3% to 1% at 24 h incubation. In more recent works, 0.3% bile is considered to be the crucial concentration to evaluate bile-tolerant probiotic LAB. From this point of view, K10, K21, and ZS07 isolates showed good resistance to bile. These findings agreed with those of studies where *L. plantarum* strains from cheeses displayed good resistance to bile salts [5, 29, 33].

With the exception of two strains (K09 and ZS11), all isolates were tolerant to sodium taurocholate (TC), sodium taurodeoxycholate (TDC), sodium glycocholate (GC), and glycodeoxycholate (GDC); some isolates were even able to deconjugate them (Table 2). Strains CK19 and ZS07 were able to grow in the presence of individual bile salts, but no bile salt deconjugation was observed. Deconjugation activity was observed for CK06, B01, K21, and ZS15 on sodium taurocholate, taurodeoxycholate, and sodium glycocholate. Deconjugation activity was not observed for all isolates on sodium glycodeoxycholate. Deconjugation (bile salt hydrolytic-BSH activity) is another fundamental property for probiotic strains to survive the toxicity of conjugated bile salts in the duodenum. In our work, conjugated salts of cholic acid were more toxic than those of deoxycholic acid, and salts of glycine were also more inhibitory than salts of taurine. These results are in agreement with previous data reported by other authors for lactobacilli and bifidobacteria [35–39].

Most *L. plantarum* isolates showed detectable deconjugation of primary bile salts GC and TC in the conditions of the experiment. Furthermore, eight of the tested isolates effectively deconjugated secondary salts (TDC). Previous works of [29, 33] also indicated that *L. plantarum* strains were able to deconjugate bile salts. The fact that some strains were able to grow in the presence of conjugated bile salts, while they were not able to deconjugate them, is in accordance with the hypothesis that the capacity to express bile salt hydrolase is not related to the capacity to resist the toxicity of conjugated bile salts [40].

*β*-galactosidase activity was present in all strains (Table 2). The values of *β*-galactosidase activity ranged from 24 to 1925 Miller units. The highest values, ranging from 1024 to 1925 Miller units, were obtained for five isolates (LM11, ZS07, CK06, B01, and ZS11). A low level of *β*-galactosidase was produced by other isolates (92–213 Miller units). The lowest activity value was obtained for K2 (24 Miller units). *β*-galactosidase activity is an essential feature in probiotic strains. Lactose intolerance (*β*-galactosidase deficiency) is linked to the inability to break down lactose in the upper regions of the small intestine, which is thus utilized by the indigenous microbiota [41]. The values found for the tested *L. plantarum* isolates are in the range of values previously reported [29]. Very high *β*-galactosidase activity was detected in five tested *L. plantarum* strains, which might therefore be used as a dietary adjunct to moderate lactose intolerance in the gut.

Phenotypic antibiotic resistance was investigated by MIC analysis, using the microbiological cutoff values for ampicillin, gentamicin, kanamycin, erythromycin, clindamycin, tetracycline, and chloramphenicol reported by EFSA document [15] for *L. plantarum* to distinguish between susceptible and resistant strains. The results obtained for antibiotic resistance of the 11 isolates from Bryndza cheese are shown in Table 2. All strains of *L. plantarum *isolates were susceptible to ampicillin, gentamicin, tetracycline, and chloramphenicol. A high percentage of isolates (63.6%) was resistant to kanamycin. Only three isolates were resistant to erythromycin and one isolate to clindamycin. Flórez et al. [42] identified nearly all 121 *L. plantarum* strains of plant and dairy origin to be susceptible to ampicillin, clindamycin, erythromycin, and gentamicin. Resistance against kanamycin has been observed more frequently among lactobacilli [43]. Pinto et al. [44] examined the susceptibility of *L. plantarum* from traditional African fermented products and found susceptibility to ampicillin, erythromycin, tetracycline and chloramphenicol, in agreement with our study except erythromycin. Furthermore, they detected gentamicin resistance, in contrast to our results. However, Zago et al. [29] detected all 27 *L. plantarum* isolates from cheeses susceptible to gentamicin. Contrary to the findings of Zonenschain et al. [45], who found a high frequency of tetracycline-resistant strains in *L. plantarum* from Italian fermented dry sausages, we observed only susceptible isolates. All strains under study did not contain the transferable, acquired resistances toward tetracycline and chloramphenicol, but resistance to erythromycin was confirmed in three out of 11 isolates.

Eleven *L. plantarum* were investigated for their potential to form histamine, tyramine, and putrescine using the qualitative assay in a decarboxylase medium. No isolate was able to form histamine and putrescine. CK06, LM11, and ZS11 were found to decarboxylate tyrosine into the respective biogenic amine tyramine. Three out of 11 *L. plantarum* tested in present study were able to decarboxylate tyrosine into tyramine. This result is consistent with published data [16, 46, 47], which show that tyramine is the most common amine associated with growth of lactic acid bacteria mainly belonging to the genera *Enterococcus* and *Lactobacillus* from different sources such as wine, meat, and cheeses. Also other amines, such as histamine, cadaverine, putrescine, tryptamine, and phenylethylamine, have been found in many types of fermented products [48–50]. This study showed that most of the tested *L. plantarum* isolates do not have the ability to form these biogenic amines. Because the production of BAs by lactic acid bacteria is not a desirable property, only the amine-negative isolates are suitable when selecting strains as probiotics, dietary adjuncts, and starter cultures.

## 4. Conclusions

In conclusion, the screening of acid-resistant lactobacilli strains from Slovak Bryndza cheese resulted in isolation of new active isolates identified as *L. plantarum*. Strains ZS07 and K21 showed positive traits (antibacterial activity, acid resistance, and safe activity), which gives them a good probiotic potential. Some additional studies should be done to know the power of adhesion and the stability of the strains to manufacturing processes. The *in vitro* screening of lactobacilli from Slovak Bryndza Cheese constitutes a valuable strategy for the large-scale preliminary selection of putatively safe LAB intended for use as probiotic cultures.

## Figures and Tables

**Table 1:** Numbers of predictive lactobacilli isolates with antimicrobial effect against indicator strains.

Inhibition zone	LM	SA	SE	SL	EF	SAE	SP	AC
1–5 mm	116	107	99	102	100	116	84	64
<1 mm	9	18	26	23	25	9	41	61

LM: *Listeria monocytogenes*; SA: *Staphylococcus aureus*; SE: *S. epidermidis*; SL: *S. lentus*; EF: *Enterococcus faecalis*; SAE: *Salmonella enterica*; SP: *Sphingomonas paucimobilis*; AC: *Acinetobacter calcoaceticus*.

**Table 2:** Survival of Bryndza cheese isolates in the presence of 0.3%, 0.5%, and 1% bile salts, deconjugation of bile salts, *β*-galactosidase activity, and antibiotic susceptibility.

Strain	Growth (%) in the presence of bile (mean ± SD)			Deconjugation of bile salts				*β*-galactosidase activity (Miller units, mean ± SD)	Minimum inhibitory concentrations (mg/L) of the antibiotics						
	0.3%	0.5%	1.0%	TC	TDC	GC	GDC		AMP	GEN	KAN	ERY	CM	TET	CMP
CK06	31.29 ± 4.54	11.08 ± 3.44	2.80 ± 1.30	d	sd	sd	g	1620.77 ± 141.70	0.25	2	**128**	**8**	0.06	8	2
CK19	28.91 ± 8.33	29.26 ± 6.77	18.62 ± 6.77	g	g	g	g	169.50 ± 35.20	0.5	4	**128**	**4**	**4**	32	4
B01	35.58 ± 12.37	13.40 ± 1.10	7.73 ± 3.64	sd	d	d	g	1725.28 ± 233.90	2	4	**128**	0.25	0.06	1	1
B07	42.31 ± 12.74	10.44 ± 5.44	5.76 ± 1.14	g	g	sd	g	92.34 ± 8.80	0.25	4	**128**	0.5	0.06	2	4
K09	30.78 ± 12.75	15.31 ± 3.61	0.00	g	d	wg	g	120.79 ± 29.20	1	8	64	0.5	0.12	32	2
K10	53.10 ± 7.28	12.28 ± 1.24	8.77 ± 3.72	g	d	d	g	144.82 ± 37.60	0.25	4	**128**	0.25	0.03	8	2
K21	51.45 ± 11.10	21.76 ± 9.20	11.76 ± 1.50	sd	sd	d	g	23.89 ± 2.90	0.25	2	64	0.12	0.06	4	1
LM11	29.26 ± 6.70	15.97 ± 0.44	11.76 ± 1.34	g	sd	g	g	1024.38 ± 74.40	1	2	**128**	**8**	2	8	4
ZS07	65.35 ± 12.47	25.52 ± 8.41	18.23 ± 3.68	g	g	g	g	1365.85 ± 70.90	0.25	1	**128**	0.12	0.03	1	1
ZS11	21.34 ± 2.82	10.91 ± 2.57	6.36 ± 3.00	d	d	wg	g	1925.28 ± 203.10	0.25	0.5	32	1	0.03	16	4
ZS15	28.41 ± 6.85	11.29 ± 5.32	12.37 ± 1.52	d	sd	d	g	213.84 ± 18.00	0.25	1	32	0.5	0.03	16	1

TC: sodium salts of taurocholic acid; TDC: taurodeoxycholic acid; GC: glycocholic acid; GDC: glycodeoxycholic acid; d: deconjugation; sd: strong deconjugation; g: growth; wg: weak growth.
